# A nationwide survey of the influence of month of birth on the risk of developing multiple sclerosis in Sweden and Iceland

**DOI:** 10.1007/s00415-017-8665-y

**Published:** 2017-11-20

**Authors:** Olöf Eliasdottir, Anders Hildeman, Marco Longfils, O. Nerman, J. Lycke

**Affiliations:** 1 Department of Clinical Neuroscience, Institute of Neuroscience and Physiology, Sahlgrenska Academy, University of Gothenburg, Blåa stråket 7, 41345 Gothenburg, Sweden; 2 Department of Mathematical Sciences, Chalmers University of Technology and University of Gothenburg, Gothenburg, Sweden

**Keywords:** Multiple sclerosis, Month of birth, Risk factors, Epidemiology, Sweden, Iceland

## Abstract

Previous studies have shown that the risk of multiple sclerosis (MS) is associated with season of birth with a higher proportion of MS patients being born in spring. However, this relationship has recently been questioned and may be due to confounding factors. Our aim was to assess the influence from season or month of birth on the risk of developing MS in Sweden and Iceland. Information about month of birth, gender, and phenotype of MS for patients born 1940–1996 was retrieved from the Swedish MS registry (SMSR), and their place of birth was retrieved from the Swedish Total Population Registry (TPR). The corresponding information was retrieved from medical journals of Icelandic MS patients born 1981–1996. The control groups consisted of every person born in Sweden 1940–1996, their gender and county of birth (TPR), and in Iceland all persons born between 1981 and 1996 and their gender (Statistics Iceland). We calculated the expected number of MS patients born during each season and in every month and compared it with the observed number. Adjustments were made for gender, birth year, and county of birth. We included 12,020 Swedish and 108 Icelandic MS patients in the analyses. There was no significant difference between expected and observed MS births related to season or month of birth in Sweden or Iceland. This was even the results before adjustments were made for birth year and birth place. No significant differences were found in subgroup analyses including data of latitude of birth, gender, clinical phenotype, and MS onset of 30 years or less. Our results do not support the previously reported association between season or month of birth and MS risk. Analysis of birth place and birth year as possible confounding factors showed no major influence of them on the seasonal MS risk in Sweden and Iceland.

**Electronic supplementary material:**

The online version of this article (10.1007/s00415-017-8665-y) contains supplementary material, which is available to authorized users.

## Introduction

There is accumulating evidence that implies low sun exposure and low levels of vitamin D as risk factors for multiple sclerosis (MS) [13, 15, 17]. This association may explain the increasing incidence and prevalence of MS observed with the distance from the equator [3, 20]. In fact, a gradient of increased MS prevalence with north latitude has also been reported in Sweden [1]. Moreover, low ultraviolet radiation of pregnant women during winter has been a reasonable explanation for the increased risk of MS observed in persons born during spring and the reduced risk in those born during winter [26]. The changed risk of MS related to month of birth (MOB) has also been reported in the Scandinavian countries [10, 16, 21] including Sweden [22]. However, this relationship has recently been questioned, and confounding factors rather than biology were suggested to generate the association between MOB and MS risk [7, 8]. The highly variable birth rate, which is influenced by birth year and regional (birth county) variations, may be responsible for the previous findings [7, 8]. Although a recent study of Norway did take these confounding factors into account for, they claimed an increased MS risk in persons born in April [25]. In light of this, we conducted a study of association between season or MOB and the risk for developing MS in Sweden and Iceland. Our aim was to compare our results with those previously published on MOB and MS risk from other populations and to clarify the effect from birth year and birth place as confounding factors.

## Materials and methods

The study was based on two nationwide population cohorts of Sweden and Iceland. All patients had clinically definite or clinically probable MS according to Poser diagnostic criteria or MS according to the revised McDonalds criteria [18, 19].

### Area and population

Sweden lies between latitudes 55° and 69° north in Northern Europe. There are 290 municipalities in Sweden. The population density is considerably higher in the southern part of the country. During the study period from 1940 to 1996, the Swedish population increased from 6.4 to 8.8 million people, the mean age increased from 37.0 years in 1968 (the first year of registration of mean age) to 39.7 years, the birth rate per 1000 decreased from 15.1 to 10.8, and the mortality per 1000 decreased from 11.4 to 10.6. (http://www.scb.se).

Iceland lies between latitudes 64° and 66°N. The population of Iceland increased between 1981 and 1996 from 0.23 to 0.27 million people, the mean age increased from 31.6 to 33.9 years, the birth rate per 1000 decreased from 19.0 to 16.2, and the mortality rate per 1000 decreased from 7.2 to 7.0. In 1996, approximately 70% of the population lived in Reykjavik, the capitol of Iceland which lies at 64°N (http://www.statice.is).

### The Swedish registries

The Swedish MS registry (SMSR), started in 1996, became web based 2004, and 2008 included 14,500 of Sweden’s estimated 17,500 prevalence patients, giving coverage of 80% [1, 11]. The registry serves as a national quality health care registry for Swedish MS patients. Patients with MS according to the Poser [19] or the McDonald criteria [18] have been prospectively or retrospectively registered (http://www.neuroreg.se).

The Swedish Total Population Registry (TPR), founded in 1947, registers residence over time for all residents in Sweden with some retrospectivity (http://www.scb.se). Before 1961, there was a chance that individuals were not included in the registry if they lived unmarried together with another person and without children; however, thereafter, the registry is complete [14].

### The Swedish MS cohort

MS patients included in the study were born in Sweden between 1940 and 1996. This period was chosen to decrease the effect of decease bias for patients born before 1940 and the possibility that patients born after 1996 might not yet have developed the disease. Deceased patients were included. At 31 January 2016, the day of data export, the following data were retrieved from the SMSR for every patient: personal identity number, month, season and year of birth, gender, date of MS onset, age at MS onset, age at data export, MS phenotype (relapsing–remitting (RRMS), secondary progressive (SPMS), progressive relapsing MS (PRMS), and primary progressive MS (PPMS), date when patients reach Expanded Disability Status Score (EDSS) 6 [12]. The following data were retrieved from the TPR: place of birth.

### The Swedish control cohort

We created a control group (*n* = 3,503,550) of every person born in Sweden 1940–1996, their gender and county of birth according to TPR.

### The Icelandic MS cohort

The Icelandic MS patients were born in Iceland 1981–1996. There are no data available for persons born prior to 1981 in Iceland. Identical data as for the Swedish MS cohort were retrieved for Icelandic MS patients.

The patients were retrieved from different sources to make the cohort as complete as possible. The diagnosis searched for was: ICD10 (G35, G37.9), ICD9 (340, 341), and ICD8 (340, 341).

1. The neurology department at the Landspitali University Hospital; the only university hospital in Iceland which handles referrals for the whole country. Information was retrieved for both inpatients and outpatients.
2. All private practicing neurologists in Iceland.
3. Smaller hospitals and rehabilitation centers.
4. All patients approved for treatment of MS with disease modifying therapies, i.e., treatments in need of approval by a centralized agency.
5. Information from the Icelandic Social Security Agency to identify all who received disability benefits in Iceland.

### The Icelandic control cohort

We created a control group (*n* = 65,114) from the Icelandic population born 1981–1996, divided according to gender (http://www.statice.is).

### Statistical methods

All tests were based on the observed numbers of births in a certain season or month. These observations were compared to adjusted expected means. These adjusted means where derived from a simple Bernoulli distribution for each case with probabilities equal to the relative frequency of births in the MS case specific stratum (gender, birth year, and county). A central limit argument implies that we can use normal approximation for the null hypothesis reference distributions. By assuming independence between all Bernoulli distributions, we receive marginally larger variance than we would have got by taking multiple case correlations inside the strata into account.

Primarily, we look for over-representations, but we also acknowledge under-representations, if they would show up, using two-sided tests. The statistical approach was originally to test for seasonal effects [(March, April, May), (June, July, August), (September, October, November), and (December, January, February)]. When we did not find any signs of effects, there we started an exploratory phase of testing analyzing gender divided data, months instead of season, specific MS subgroups including early and late onset groups, and geographical north–south separation. Certainly, multiple correction problems arise in this second step. However, the purpose of this step was partly to enable comparisons with findings in the Norwegian study [25] and confirm the negative findings in the first step. For more arguments behind this approach, cf. the discussion section below.

A normal test was used with the null hypothesis that there is no difference in the probability of getting MS for male or female depending on the birth date. This null hypothesis was tested against an alternative hypothesis that there is an increased risk getting the disease in a certain period. We used the Bernoulli estimation model to calculate the expected number of MS patients being born in every month and compared it with the observed number with a two-sided *T* test (*p* = 0.05). The calculations were done with and without adjustments for gender, year of birth, and county of birth as suggested by Fiddes et al. [7]. This test was also applied when the cohort was divided according to birth in Southern or Northern Sweden. We divided Sweden into two regions, a northern and a southern region, divided at the geographical middle of Sweden, i.e., 62″N. Thereafter, we calculated the observed vs expected MS MOB in the two regions. Similar tests were used for subgroup analysis related to gender, MS phenotype (RRMS, SPMS, PPMS), and early onset age (≤ 30 years of age). The statistical testing procedure is described in Supplement 1.

The calculation was done using Matlab (Mathworks, Natick, MA, USA).

The study was approved by the regional ethical review board of Gothenburg, Sweden and Icelandic National Bioethics Committee and Data Protection Authority.

## Results

### The Swedish MS cohort

We included 12,020 MS patients born in Sweden 1940–1996. The export from the MS registry was made 31st of January 2016. At that date, the SMSR included 15,801 patients, 14,157 of them were born in Sweden, 13,398 of them were born 1940–1996, and 12,020 of them had information of place of birth. The mean age of this final cohort was 51 years (median 51 years). The mean age at MS onset (*n* = 11,137) was 32.9 years (range 1–70 years). The mean age at MS diagnosis (*n* = 10,065) was 37.4 years (range 6–73 years). The female:male ratio was 2.5:1. MS phenotype was available for 11,412 patients: RRMS (*n* = 7087, 62.0%), PPMS (*n* = 932, 8.2%), PRMS (*n* = 154, 1.3%), and SPMS (*n* = 3239, 28.4%). There were 87.6% (*n* = 10,283) living in the Southern region of Sweden and 12.4% (*n* = 1458) in the Northern region of Sweden.

### The risk of MS according to season and month of birth

**Fig. 1:**
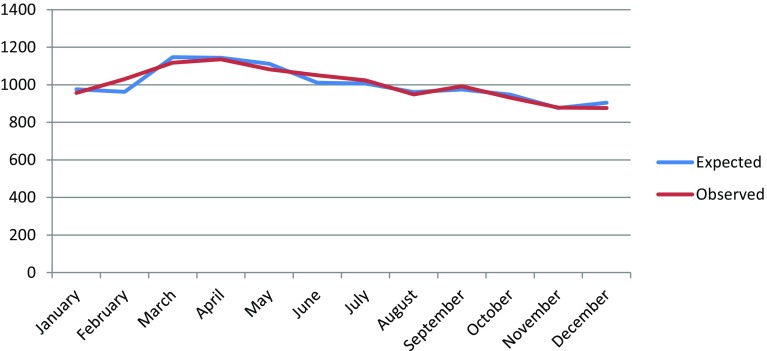
Seasonality of MS births in Sweden, with adjustments for gender, year of birth, and county of birth

We found no relationship between season of birth and the risk of developing MS later in life (Supplement 2). Neither did we find any difference between observed and expected number of MS patients when each MOB was analyzed separately (Fig. 1) with or without adjustments for birth year and county of birth. With adjustments, there seemed to be 7% more MS births in February than expected (1030 vs 961.8 *p* = 0.0208, Supplement 3). However, when Bonferroni correction is done, this difference is far from significant. To reject the null hypothesis for 5% level, we would need a *p* value of less than 0.0042 for statistical significance (0.0084 in case of one sided tests).

### The risk of MS associated with month of birth and latitude

When latitude was tested, there were 10% more MS births in February than expected in the Southern region of Sweden (900 vs 824.5, *p* = 0.00574). After Bonferroni correction for multiple comparisons, the effect was not statistically significant (Supplements 4 and 5).

### The risk of MS associated with month of birth and gender

No significant difference was found in observed vs expected births when gender was taken into account. There was a trend towards more MS births in February both in men (303 vs 276.9 *p* = 0.099) and females (727 vs 685.0 *p* = 0.0912).

### The risk of MS associated with month of birth and phenotype

No effect of MOB was seen on the risk of MS when the cohort was divided according to MS phenotype.

### The risk of MS associated with month of birth and early age of MS onset (≤ 30 years of age)

In spring, there were fewer MS births than expected (1372 vs 1442.0 *p* = 0.0285), but a *p* value of 0.0125 or lower would have been needed to reach statistical significance after correction for multiple tests (Supplement 6). Nor did we find any significant differences in the number of MS births related to month.

### The Icelandic MS cohort

At the 01st of January 2016, we included 108 patients born in Iceland from 1981 to 1996. The mean age of the cohort was 30.1 years (range 20–35 years), the mean age at MS onset was 22.5 years (range 10–37 years), and the mean age at MS diagnosis was 23.7 years (range 13–39 years). The female:male ratio was 2.1:1.

### The risk of MS according to season and month of birth

We found no relationship between season of birth and the risk of developing MS later in life (Supplement 7). Neither did we find any difference between expected and observed number of Icelandic MS patients when each MOB was analyzed separately (Supplement 8). In our original analysis, there seemed to be fewer MS births in the autumn (17.0 vs 26.9, *p* = 0.028). After correction for multiple tests, this effect disappeared.

## Discussion

Our study showed no influence from season of birth or MOB on the risk of developing MS later in life in Sweden or Iceland with or without adjustments for possible confounding factors [7]. Even in the subgroup analysis, no observations remained as statistically significant after correction for multiple hypothesis testing, which confirms the negative result in the seasonal analysis. The only month that showed higher MS births was February, where we observed 7% more MS cases than expected. This corresponds to a *p* value of 2.08% before any multiple corrections. This significance disappeared after correction for multiple testing. Although low vitamin D levels during fetal development might be a risk factor for developing MS later in life [6], our results did not support that this risk is associated with season of birth or MOB.

In a recent study from Norway, including a cohort of 6649 MS patients born 1930–1979 [25], they found a 10% increase in MS births in April, a 15% increase in December, and, in contrast with our study, a 13% decrease in February before any cofounding factors adjustments. However, after correction for birth year, the increase in December disappeared, and after correction for birth county, the decrease in February became non-significant. However, a 10% increase in birth of the Norwegian MS patients remained in April, and this increase was also significant when comparing the incidence of MS in siblings, mothers, and fathers. Although the authors concluded that there was an increased risk in April births in the MS population, the significance disappeared after correction for multiple testing. We showed only marginally smaller observed MS cases in April.

We also found that the MS incidence was higher than expected in February in the Southern region of Sweden. The *p* value is 0.5% but not significant after correction for months. Moreover, this association was not in line with the previous hypothesis, suggesting an increased MS risk with northern latitude, less sun exposure and low D vitamin levels.

In contrast with our results, a previous study from Sweden showed more cases of MS than expected in June (11%) and fewer than expected in December and January (8 and 10%), respectively [22]. Although their MS patients (*n* = 9461) were also retrieved from the SMSR registry, their study population and controls differed from ours in several aspects. Their patients had a median birth year of 1957 and included all patients registered in the SMSR until 2008. Our study cohort was younger with a median birth year of 1965, and included only patients born between 1940 and 1996 to decrease the effect of decease bias for patients born before 1940 and the possibility that patients born after 1996 might not yet have developed MS. However, even with our study design, we might have missed patients born after 1940 who due to severe MS died before 1996 when the SMSR was established. This limitation in year of birth influenced the size of the control cohort, and neither did we include controls from municipalities that did not have a case of MS. Another factor that should be noted is the change of MOB of patients with MS over recent years. In contrast with the previous investigation [22], we found no increased MS risk related to birth in June in patients registered after 2008. Moreover, no correction for year of birth or county of birth was made when analyzing the MS risk in that study. They used 2 × 2 Chi-squared test for calculating MOB and MS risk. Applying that test to our data for a direct comparison did not influence our results.

We searched PubMed and Scopus for studies investigating the possible influence from season of birth or MOB on the risk of developing MS (Table 1). Except for the Norwegian study [25], other studies have not adjusted the results for birth year and birth place. They have made adjustments for either birth place [2, 4, 5, 24], or birth year [21], or none of these confounding factors [26]. However, without adjustments, similar result as we found was found in a South African study [9] and in a study from Portugal with 1207 MS patients and 1207 match controls, after adjusting the material for latitude of birth and gender [4]. However, birth year was not taken into account in this analysis. In all other studies, an association has been showed between increased MS risk and birth during spring and/or a decreased MS risk in persons born during autumn or winter [5, 21–24, 26] or the opposite in a study from the Southern hemisphere [2].

**Table 1:** Owerview over studies of birth month and MS risk

Country	Author	Publication year	Birth years of MS group	Included patients (n)	Controls	Main findings	Adjustment for birth year/birthplace
Canada, Great Britain, Denmark, Sweden [26]	Willer et al.	2005	1926–1970 (Canada)	17,874, 11,502, 6276, 6393 (total 42,045)	13,675,451 (Canada)	9.1% more in May, 8.5% fewer in November	No/no
Sweden [21]	Salzer J et al.	2010	1900–2007	9361	12,116,853	11% more in June, 8 and 10% fewer in December and January	No/no
Italy [23]	Sotgiu et al.	2006	nd^a^	810	247,612	More births in spring months	No/no
Scotland [5]	Bayes et al.	2010	1922–1992	1309	6,198,352	17% more in spring, 13% fewer in autumn	No/yes
Australia [24]	Staples et al.	2010	1920–1950	1524	2,468,779	1.34 risk for those born in November–December compared May–June^b^	No/yes
Kuwait [2]	Akhtar et al.	2015	1950–2013	1035	3,454,222	13% more in December	No/yes
Portugal [4]	Barros et al.	2013	1992–1943	421	1,150,362	No seasonal difference	No/yes
Norway [25]	Torkildsen et al.	2014	1930–1979	6649	2,899,260	No seasonal difference	Yes/yes
Finland [21]	Saastamoinen et al.	2012	1900–1988	8739	7,014,435	9.4% more in April, 11.1% fewer in November	Yes/no
South America [9]	Fragoso et al.	2013	nd^a^	1207	1207	No seasonal difference	Yes/yes

^a^Not defined

^b^Incidence rate ratio for two-month period with May–June as the reference period (1.0)

The main strength of our study is that data was retrieved from national registries with high patient recovery. The Swedish MS registry had high coverage, estimated about 80% of all cases according to the National Patient Registry (NPR) at the National Board of Health and Welfare (http://www.socialstyrelsen.se) [11]. In a previous study, we found that the rate of older MS patients might be lower in the SMSR than in the NPR, while early MS cases are almost completely included, but there might be regional variation in the inclusion rate [1]. However, these differences should not have influenced the result of our study. The Icelandic data were population based and probably included all MS patients in Iceland. Although we did not remove MS cases from the general Icelandic population, this should have minimal effect on the results due to the small number.

In conclusion, our results did not support the previously reported association between season or MOB and MS risk. Our results were unaffected by adjustments for possible confounding factors, and therefore, it remains unclear if those are responsible for the previously reported relationship between birth during spring and an increased MS risk [7]. Thus, our results do not support the hypothesis that pregnancy during autumn and winter with low levels of sun exposure and low vitamin D levels influence the risk of MS.

## Electronic supplementary material

Below is the link to the electronic supplementary material.

Supplementary material 1 (DOCX 15 kb)

Supplementary material 2 (DOCX 23 kb)
