# Supplementary material for: A nationwide survey of the influence of month of birth on the risk of developing multiple sclerosis in Sweden and Iceland
Source: J Neurol. 2017 Nov 20;265(1):108–14. doi: 10.1007/s00415-017-8665-y (PMC5760596; doi:10.1007/s00415-017-8665-y)
Supplement: Supplementary file 1 — Supplementary material 1 (DOCX 15 kb) [file 415_2017_8665_MOESM1_ESM.docx]

# **Supplement 1**

The testing procedure used in this article is as follows.

Consider the null hypothesis that there is no difference in the probability of getting MS depending on the birth month/season (inside a given community at a given year of birth for a given sex). This null-hypothesis is tested against the alternative hypothesis that there is a difference.

The test uses a sum of simple statistics each pertaining to a diseased individual. These basic statistics are 0-1 valued random variables indicating with 1 if the individual is born in the season (month) in question and with 0 if not, and each of them are, because of the equal probability assumption, conditionally Bernoulli distributed with probability p = the proportion of the individuals in the stratum born in the season, given the birth pattern of all individuals in the stratum.

So for each *i*, where i indicates the stratum (year, municipality, sex), we know the number of patients born, N_i_. For the control group in *i*, the probability of being born in season *j* is denoted as p_ij_. If we condition on the whole birth date pattern, p_ij_ = M_ij_/M_i_ will be the conditional probability under the null hypothesis that the particular case is born in season *j*. Here Mi is the total number of controls born in *i* and M_ij_ is the converse for *i* and the particular season *j*.

According to the null hypothesis, the patients births should also be distributed according to p_ij_. This means that by knowing N_i_ and pij we can estimate the expected number of people with MS in the stratum *i* and season *j* as $\hat{N}$_ij_ = N_i_p_ij_. The difference between the observed and expected number of patients in each stratum and month we denote by X_ij_ = N_ij_ - $\hat{N}$_ij_. The variance of X_ij_ can be estimated as V_ij_ = N_i_ p_ij_(1-p_ij_). Where V_ij_ explain how much the observed patient births N_ij_ are expected to vary around $\hat{N}$_ij_.

The test statistic $T_{j}=\frac{\sum_{i} X_{ij}}{\sum_{i} V_{ij}}$ is adopted and we use a normal approximation to the null hypothesis distribution assuming independence between all the contributing case indicators to derive p-values. The normal approximation works since the variance of each X_ij_ are small compared to the total sum $\sum_{i} V_{ij}$

Large positive values of T_j_ corresponds to more observed patients than expected in season *j*. Analogously, large negative values correponded to less observed patients than expected in season *j*.
