# Supplementary material for: A nationwide survey of the influence of month of birth on the risk of developing multiple sclerosis in Sweden and Iceland
Source: J Neurol. 2017 Nov 20;265(1):108–14. doi: 10.1007/s00415-017-8665-y (PMC5760596; doi:10.1007/s00415-017-8665-y)
Supplement: Supplementary file 2 — Supplementary material 2 (DOCX 23 kb) [file 415_2017_8665_MOESM2_ESM.docx]

| Supplement 2 Observed and expected MS births in Sweden after different seasons (n=12019) | | | | | | | | |
| --- | --- | --- | --- | --- | --- | --- | --- | --- |
| **Season** | **Spring*** | **Summer**** | **Autumn^Ψ^** | **Winter^ϯ^** |  |  |  |  |
| **p-values†** | 0.156 | 0.396 | 0.97 | 0.497 |  |  |  |  |
| **Expected** | 3403.4 | 2982.1 | 2803.7 | 2829.7 |  |  |  |  |
| **Observed** | 3334 | 3022 | 2802 | 2861 |  |  |  |  |
| *March, April, May **June, July, August Ψseptember, October, November ϯ December, January, February | | | | |  |  |  |  |
| † Adjusted for gender, year of birth and county of birth | | | | | | | | |

| Supplement 3 Observed and expected MS births in Sweden (n=12020). | | | | | | | | | |
| --- | --- | --- | --- | --- | --- | --- | --- | --- | --- |
|  |  |  |  |  |  |  |  |  |  |
| **Month** | **January** | **February** | **Mars** | **April** | **May** | **June** | **July** | **August** | **December** |
| **p-values†** | 0.496 | 0.0208 | 0.356 | 0.804 | 0.343 | 0.194 | 0.568 | 0.677 | 0.332 |
| **Expected** | 976.2 | 961.8 | 1146.5 | 1142.9 | 1111.9 | 1010.8 | 1005.8 | 961.3 | 903.8 |
| **Observed** | 956 | 1030 | 1117 | 1135 | 1082 | 1050 | 1023 | 949 | 876 |
| † Adjusted for gender, year of birth and county of birth | | | | |  |  |  |  |  |

| Supplement 4 Observed and expected MS births in North-Sweden (n=1458) | | | | | | | | | |
| --- | --- | --- | --- | --- | --- | --- | --- | --- | --- |
|  |  |  |  |  |  |  |  |  |  |
| **Month** | **January** | **February** | **Mars** | **April** | **May** | **June** | **July** | **August** | **December** |
| **p-values†** | 0.218 | 0.26 | 0.31 | 0.301 | 0.419 | 0.248 | 0.675 | 0.439 | 0.193 |
| **Expected** | 121.1 | 113.4 | 136.2 | 138.6 | 133.8 | 123.8 | 125.5 | 118.0 | 109.0 |
| **Observed** | 134 | 102 | 125 | 150 | 125 | 136 | 121 | 126 | 96 |
| † Adjusted for gender, year of birth and county of birth | | | | | | | | | |

| Supplement 5 Observed and expected MS births in South-Sweden (n=10283) | | | | | | | | | |
| --- | --- | --- | --- | --- | --- | --- | --- | --- | --- |
|  |  |  |  |  |  |  |  |  |  |
| **Month** | **January** | **February** | **Mars** | **April** | **May** | **June** | **July** | **August** | **December** |
| **p-values†** | 0.17 | 0.00574 | 0.457 | 0.51 | 0.507 | 0.263 | 0.471 | 0.501 | 0.699 |
| **Expected** | 831.6 | 824.5 | 983.0 | 977.5 | 954.4 | 864.8 | 857.9 | 823.4 | 772.3 |
| **Observed** | 794 | 900 | 961 | 958 | 935 | 896 | 878 | 805 | 762 |
| † Adjusted for gender, year of birth and county of birth | | | | | | | | | |

| Supplement 6 Subgroup analysis for debut age <30 years. Observed and expected of MS births in Sweden (n=5107) | | | | | | | | | | | | | | | | |
| --- | --- | --- | --- | --- | --- | --- | --- | --- | --- | --- | --- | --- | --- | --- | --- | --- |
|  |  |  |  |  |  |  |  |  |  |  |  |  |  |  |  |  |
| **Season** | | **Spring** | | **Summer** | | | **Autumn** | | | | **Winter** |  |  |  |  |  |
| **p-values†** | | 0.0285 | | 0.331 | | | 0.24 | | | | 0.873 |  |  |  |  |  |
| **Expected** | | 1445.1 | | 1272.1 | | | 1184.0 | | | | 1210.8 |  |  |  |  |  |
| **Observed** | | 1 372 | | 1302 | | | 1223 | | | | 1210 |  |  |  |  |  |
| † Adjusted for gender, year of birth and county of birth | | | | | | | | | | | |  |  |  |  |  |
| Supplement 7 Observed and expected MS births in Iceland after different seasons (n=108) | | | | | | | | | |  |  |  |  |  |  |  |
| **Season** | **Spring** | | **Summer** | | **Autumn** | **Winter** | |  |  |  |  |  |  |  |  |  |
| **p-values†** | 0.332 | | 0.312 | | 0.028 | 0.853 | |  |  |  |  |  |  |  |  |  |
| **Expected** | 28.6 | | 28.4 | | 26.9 | 24.2 | |  |  |  |  |  |  |  |  |  |
| **Observed** | 33 | | 33 | | 17 | 25 | |  |  |  |  |  |  |  |  |  |
| † Adjusted for gender and year of birth | | | | | |  | |  |  |  |  |  |  |  |  |  |

| Supplement 8 Observed and expected MS births in Iceland (n=108) | | | | | | | | | |
| --- | --- | --- | --- | --- | --- | --- | --- | --- | --- |
| **Month** | **January** | **February** | **Mars** | **April** | **May** | **June** | **July** | **August** | **December** |
| **p-values†** | 0.937 | 0.689 | 0.121 | 0.622 | 0.557 | 0.336 | 0.395 | 0.114 | 0.818 |
| **Expected** | 8.8 | 8.1 | 9.4 | 9.4 | 9.3 | 9.2 | 9.5 | 9.4 | 8.4 |
| **Observed** | 9 | 7 | 14 | 8 | 11 | 12 | 7 | 14 | 9 |
| † Adjusted for gender and year of birth | | | |  |  |  |  |  |  |
